# Comprehensive analysis of circadian periodic pattern in plant transcriptome

**DOI:** 10.1186/1471-2105-9-S9-S18

**Published:** 2008-08-12

**Authors:** Andrey Ptitsyn

**Affiliations:** 1 Center for Bioinformatics, Department of Microbiology, Immunology and Pathology, Colorado State University, Campus delivery 1682 Fort Collins, CO 80523, USA

## Abstract

**Background:**

Circadian rhythm is a crucial factor in orchestration of plant physiology, keeping it in synchrony with the daylight cycle. Previous studies have reported that up to 16% of plant transcriptome are circadially expressed.

**Results:**

Our studies of mammalian gene expression revealed circadian baseline oscillation in nearly 100% of genes. Here we present a comprehensive analysis of periodicity in two independent data sets. Application of the advanced algorithms and analytic approached already tested on animal data reveals oscillation in almost every gene of *Arabidopsis thaliana*.

**Conclusion:**

This study indicates an even more pervasive role of oscillation in molecular physiology of plants than previously believed. Earlier studies have dramatically underestimated the prevalence of circadian oscillation in plant gene expression.

## Introduction

A timely response and preparedness in response to the changing environmental cues are essential for life in plants and animals alike. Since plants are dependent on light for photosynthesis, a natural assumption is that circadian (i.e., approximately daily) oscillation should be an even more prominent feature of the plant gene expression than in animals. Multiple studies have reported the existence and detailed mechanism of a circadian molecular clock in plants [1-5]. Based on the studies of the model plant, *Arabidopsis thaliana*, researchers have determined that a "substantial" part of plant transcriptome cycles follows a circadian rhythm. This estimation is based on microarray experiments that search for the genes following the circadian rhythm among the entire set of transcripts that is examined by the microarray. Early attempts to identify these genes employed two-color spotted arrays, resulting in a cumbersome experimental design, or tried to minimize expenses by increasing the time span between the sample collections. The latter produced data with a very low sampling rate, which obscured the oscillation pattern in all but a few of the least noisy genes. More recent studies [1,4] used Affymetrix (Affymetrix Inc., Santa Clara) *Arabidopsis thaliana *expression arrays. These studies focused on the role of circadian oscillation in specific regulatory and signaling systems, but the entire set of data with expression profiles of more than 22,000 transcripts over two days was made available for downloading from the public databases. Although independent, both of these sets share almost identical experimental conditions and samples at the same rate of once every four hours. These features make the data easy to compare not only with one another but also to the large body of murine circadian expression data, which is also sampled every four hours over a period of two days.

In recent years, we have published a number of studies on circadian oscillation in metabolically active peripheral tissues in mice [6,7]. We have also reanalyzed and reported the discovery of circadian oscillation in a number of independent murine data sets from public sources [8]. The results of our analysis of murine circadian data are in sharp contradiction with previous reports. We were able to demonstrate circadian oscillation in not just a small number of the genes that are presumably linked to the circadian molecular clock but in all transcribed genes. Our most recent studies [9] show that circadian oscillation is traceable not only in expressed but also in genes that were previously considered silent or unexpressed. The prevailing theory reflected in the molecular biology textbooks states that 10–15% of genes cycle within a daily period and are presumably regulated by the circadian molecular clock. Molecular clocks vary significantly in details, and the genes that form the clock may be evolutionarily unrelated, but the molecular clocks of plants, mammals, and insects share the same negative feedback principle that makes oscillation self-sustaining and adjustable. We have previously established that this theory does not reflect the reality, at least in the murine model. While the basic circadian clock is active in all central and peripheral tissues, other genes show robust, noise-free oscillation, particularly those involved in supporting basic energy metabolism and not directly linked to the circadian molecular clock. Moreover, the key elements of the cell transcription machinery itself exhibit a pronounced circadian pattern in the modulation of expression of practically every gene. We now know that the entire animal transcriptome, not just a specially regulated portion, experiences circadian oscillation. However, a reasonable expectation is to find the same observation in the plant transcriptome. Plants are even more dependent on the daily change in lighting conditions. Nevertheless, the most recent studies reported only 10.4 [1] and 16% [4] of "circadially regulated" genes in the plant transcriptome. This obvious contradiction demands a uniform re-analysis of the data using advanced methodology that has been tested in multiple previous studies.

## Results and discussion

### Overview of the analysis strategy

Independent circadian studies in plants or animals rarely use exactly the same analysis pipeline. However, comparing a representative set of studies [10-15] reveals a commonality in strategy. The most typical approach starts with the normalization and scaling of microarray experiments; then the data is filtered, and only the genes that are present at least *n *times throughout the complete timeline (i.e., the exact number varies) are selected for further analysis. Some studies [16] introduced an additional filter that selects only "actively expressed" genes, i.e., genes with an expression level that is estimated above some arbitrary threshold. This much reduced subset of transcripts is subjected to the periodicity test and, in rare cases, a panel of more than one test, followed by a false discovery rate (FDR) correction. The few genes that pass the test are further analyzed to determine the phase and the amplitude of oscillation and to visualize using profile plots and heat maps. This approach produces consistent results across a number of circadian data sets from diverse origins but also shares a common set of problems. First of all, the formulation of the null hypothesis for the statistical tests is based on the assumption of an absence of periodicity, i.e., a steady line rate of transcription for the majority of the genes. This assumption is intuitive but has no foundation in biology. Second, each gene (transcript) is tested independently. On the other hand, the authors realize that that the researchers are looking for a manifestation of the same rhythm that modulates expression of different genes and that this expression is expected to be highly correlated. Another problem common for all circadian studies is that microarray expression profiles are very expensive to generate. Additionally, even the best data sets count two consecutive circadian periods at most with samples collected every four hours. Such a low sampling rate combined with a high level of stochastic noise, which is also typical for microarray estimation of gene expression, makes testing for periodicity particularly challenging.

A series of papers that we have published since 2006 have reported new algorithms for the analysis of periodicity in gene expression, including a new statistical test for periodicity, a phase classification, an application of digital signal processing, and an analysis of same-phase groups of genes as a continuous signal [8,9,17,18]. These algorithms were instrumental in the characterization of circadian expression in peripheral tissues [6,7], the discovery of a baseline oscillation in the transcript abundance of all genes [17], the discovery of alternative transcripts oscillating with a phase shift [19], and the discovery of the extra-low expression of eukaryotic genes [9]. However, all of our studies have been conducted with murine (circadian) and yeast (metabolic oscillation) models, which obviously does not include plants in the scope of our investigations. This study aims to rectify this shortcoming and determine if our previous findings of pervasive and persistent circadian oscillation in the murine transcriptome are also true for plants.

Our analysis starts with the same preprocessing normalizing and scaling microarray experiment in a time series. Then a provisional phase of oscillation is assigned to each gene. This is done before any selection or testing for periodicity; thus, a provisional or "most likely" phase can be potentially assigned to a non-oscillating noisy expression profile. However, assigning a phase does not introduce change in the data and thus does not preclude non-oscillating profiles from being filtered out in a later step. For further analysis, expression profiles are grouped into classes based on the provisionally assigned phase. In each group, profiles are joined into the phase continuum, which maximizes the statistical power in testing for periodicity and allows the application of digital filters [17]. Our methods also do not attempt the impossible, i.e., aligning all noisy profiles by peaks at particular time point. We use only as many phase classes as possible by generating an artificial cosine curve with the given length of observation, which is typically two complete daily periods, and given sampling rate, which is typically one sample taken every four hours. This strategy of analysis is applicable to a wide variety of data and has been tested on multiple data sets that were produced by collaborators at the Pennington Biomedical Research Center as well as independent data obtained from the public databases and kindly provided by the respective authors. The detailed description of the algorithms that were used in each step of the analysis is given in the Materials and Methods section.

Among the circadian gene expression data that is available from the GEO (Gene Expression Omnibus, ), only two sets have a sufficient sampling rate and use a contemporary microarray platform (i.e., Affymetrix *Arabidopsis *expression array). For convenience in this paper, these data sets will be named by the academic affiliation of the majority of the authors, i.e., Davis [1] and Warwick [4] data sets. Unfortunately, no single experiment measures gene expression in the natural, undisturbed form. Both examine which data we reanalyze to collect samples in a constant light. The idea behind such an experimental design was to isolate the genes that are regulated by the molecular clock by presuming that all other genes will experience no oscillation without environmental cues. A brief description of the experimental conditions producing these data sets is given in the Materials and Methods section.

**Figure 1:**
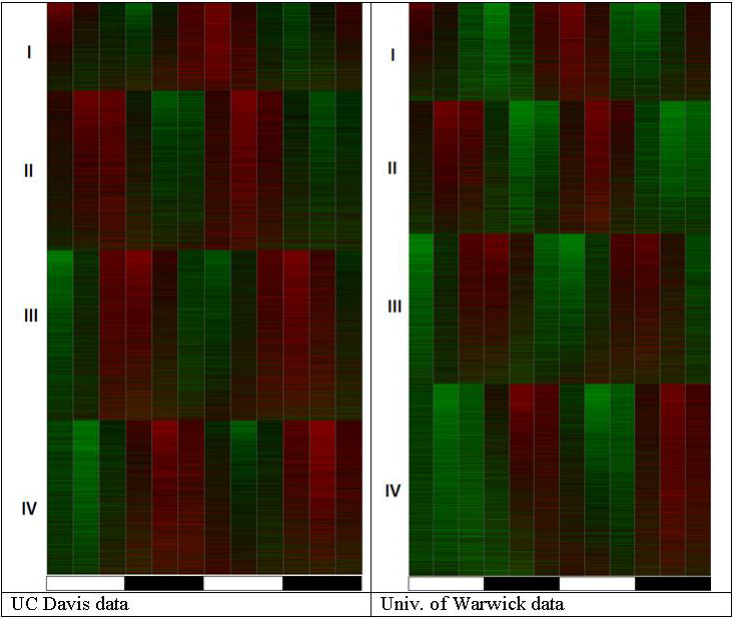
Overview of the circadian pattern of gene expression in *Arabidopsis thaliana*. All expression profiles are grouped into four separate phase classes by correlation to a cosine curve with the same sampling rate (12 points over 2 complete periods). Profiles are then sorted by decreasing autocorrelation with circadian shift. The pattern of two areas of elevated gene expression (red) interspaced by the areas of lowered gene expression (green areas) reflecting two complete cycles over two days of observation is clearly visible throughout entire data. The heatmaps show all transcripts represented on microarray (over 22,000 probesets).

Our results show that the efforts on the part of the original authors to isolate a small number of genes did not result in the intended outcome. In both data sets, the baseline circadian oscillation is statistically significant and visually detectable in practically 100% of all genes. The overview of the patterns dominating gene expression in plants is given in Figure 1. A straightforward application of a Pt-test [8] to one gene at a time identifies 8,639 transcripts (i.e., ~39% of all genes that were examined by the microarray) as oscillating in the Davis data set and 10,001 transcripts (i.e., ~44%) as oscillating in the Warwick data set with the p-value cutoff at 0.05. A less noise-tolerant autocorrelation method identifies circadian oscillation in 3,351 transcripts (i.e., ~15%) in the Davis data set and 4,324 (i.e., ~19%) in the Warwick data set. An application of the Fisher's g-test in the same setting with the same significance cutoff identifies 3,497 (i.e., ~15%) in the Davis data set and 3,918 (i.e., ~17%) in the Warwick data set. Not surprisingly, the Pt-test, which was specifically developed for a short time series with a low sampling rate, outperforms the other algorithms. However, the older algorithms report the numbers of rhythmic transcripts that are generally in agreement with those published by Edwards et al. [4], i.e., 3,505 or approximately 15% of all transcripts that are represented on the microarray. Using the same COSOPT approach [20], Covington and Harmer [1] have identified only 1,610 rhythmic transcripts (i.e., ~7%) in the Davis data set with the same significance cutoff. On one hand, this is consistent with the general observation that all methods tend to identify more rhythmic transcripts in the Warwick data set, which is probably because it has better general signal to noise ratio across all probe sets. On the other hand, such a sharp drop in the number of identified rhythmic transcripts as compared to the algorithms that are applied in this paper may indicate low robustness in the cosine curve fitting in the time domain that is employed by COSOPT; i.e., a little extra noise causes a large decrease in performance. This was one of the motivations for the development of the permutation test for periodicity, which considers only one peak in frequency domain (i.e., a periodogram), thereby making the algorithm more tolerable to noise that contributes to all other peaks [8].

**Figure 2:**
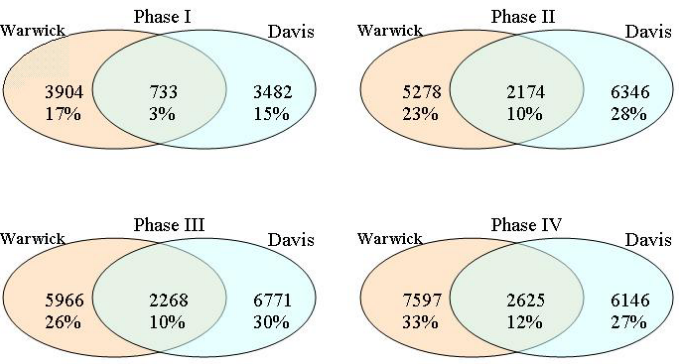
Overlap in phase of oscillation between two plant circadian data sets. While practically all genes have baseline oscillation, relatively few genes (34%) are found oscillating in the same phase between Warwick and Davis data sets. The diagram shows the absolute number (above) and percentage (rounded up, below) of transcripts oscillating in the same (overlapping area) and in different phases in each phase of four phase groups.

In spite of the similarities in the experimental conditions, relatively few transcripts are identified as oscillating in the same phase between the two data sets. The diagram of the overlapping phase groups is presented in Figure 2 and Table 1. Roughly two thirds of all transcripts are out of sync between the Warwick and Davis experiments, probably reflecting some minor differences in the environment. These genes are also possibly less important for the plant response to environmental cues and are not directly linked to the circadian clock while still modulated by oscillation in other genes. However, this hypothesis should be corroborated by further studies. This observation is consistent with previous observations in mouse gene expression: phase of expression is volatile and often varies between tissues and experimental conditions [6,8,18]. On the other hand, low sampling rate of microarray experiments also curbs the precision of phase assignment.

**Table 1:** Difference between phase groups in Davis and Warwick data sets.

		**Warwick data set**			
		
		**Phase I**	**Phase II**	**Phase III**	**Phase IV**
**Davis data set**	**Phase I**	733 3%	518 2%	607 3%	1624 7%
	**Phase II**	1450 6%	2174 10%	1242 5%	1480 7%
	**Phase III**	904 4%	1732 8%	2268 10%	1867 8%
	**Phase IV**	817 4%	854 4%	1849 8%	2625 12%

Diagonal cells show the number and percentage (rounded up) of transcripts found in the same phase in both data sets. Cells off the diagonal show the number and percentage of transcripts oscillating in different phases.

**Figure 3:**
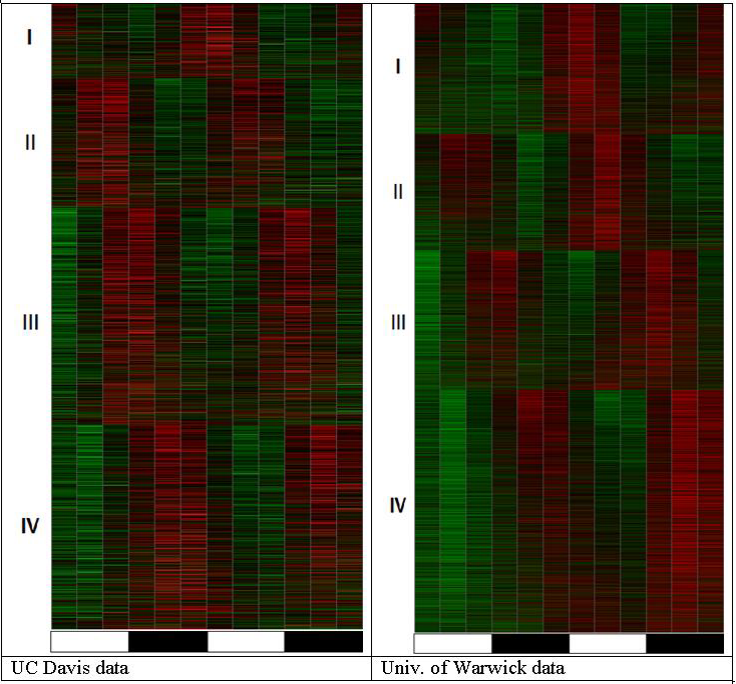
Overview of circadian oscillation pattern in genes with "absent" call. The heatmaps are produced using the same algorithm as for Figure 1. However, only the probesets called "absent" at all 12 time points are considered. In spite of being expressed below the estimated noise level in each microarray experiment, these transcripts show the same circadian expression pattern (two elevated expression periods spaced by two lower expression periods over two days) as conventionally detectable transcripts.

Results of the analysis in a classic "one gene at a time" approach are generally in agreement with each other and seem to reflect some of the patterns in the data. However, all of these methods are in acute contradiction with the results that are presented in Figure 1. The pattern in Figure 1 shows exactly two red areas of elevated transcript abundance interspaced with two green areas of lower transcript abundance, and this pattern does not stop on a fraction of 7%, 15%, or even 44%; it involves all or nearly all of the genes. The phase continuum approach [17] applies statistical testing to separate rhythmic transcripts from stochastic ones. This analysis shows remarkable agreement with the intuitively detected pattern but relies on quantitative methodology. In both the Davis and Warwick data sets, this method reported a detectable baseline circadian oscillation in 100% of all transcripts. This number does not exclude any transcript, not even those never considered present or expressed by GC-RMA (i.e., Warwick) [21] or Affymetrix MAS5.0 algorithms. Such rhythmic behavior of the "non-present" ghost transcripts has been recently reported based on studies of animal and yeast data [9] and is also supported by experimental studies [22]. Genes that are expressed below the resolution ability (i.e., presence call) for the current microarray technology are not silent; they are expressed at a low level but respond to the changing cellular and external factors. Additionally, they interact with the other related genes in biological pathways. This study confirms that the same is true for plant genes. A separate view of the oscillating pattern in low-expressed (i.e., not called present) genes is depicted in Figure 3.

**Figure 4:**
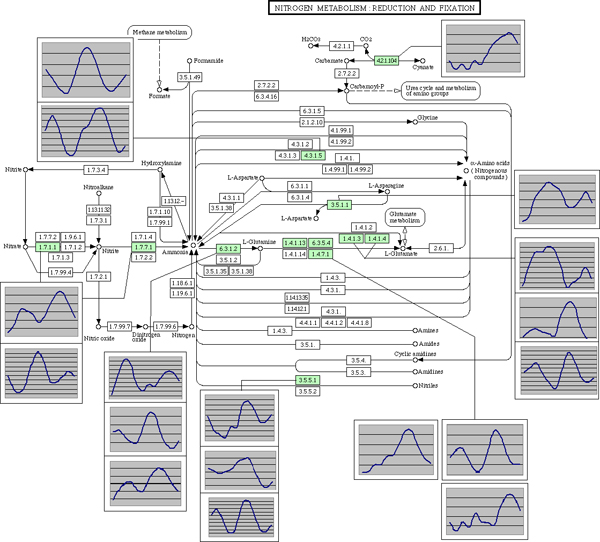
Circadian oscillation in nitrogen metabolism pathway. Expression profiles of microarray probesets are overlapped with the Kyoto Encyclopedia of Genes and Genomes (KEGG) map of the nitrogen metabolism in *Arabidopsis thaliana*. Some genes are represented by more than one set of alternative probes. Expression of the NIA2 (Nitrate Reductase 2, EC:1.7.1.1) at the start of the cascade shows the evidence of alternative probes (and thus most likely alternative transcripts) oscillating in counter-phase. Green color marks the elements of the pathway for which KEGG has additional information accessible by a clickable link and for which Affymetrix probesets are traced in the Davis data set.

Because oscillation is so pervasive, it affects not some but all biological pathways. The nitrogen reduction metabolic pathway, which is shown in Figure 4, provides an example. The known components of the pathway are traced to the probe sets in the Davis data, and their expression profiles are overlapped with the KEGG pathway map [23]. Even though the Davis data is noisier, a circadian oscillation pattern with two humps over two days of observation is apparent in most expression profiles. Remarkably, a few components of the nitrogen reduction pathway have alternative probe sets that oscillate with a phase shift or directly in counter-phase to each other. This phenomenon has already been reported with animal data [19] as a possible molecular mechanism compensating for constant oscillation and creating a steady transcript abundance over time, thus providing a steady translation rate and stabilizing the volume of signal transduction at any time of the day. A similar pattern of expression in alternative probe sets for the same gene may have a similar explanation as in *Arabidopsis*. A steady abundance of the transcripts that is created by alternating transcripts with a different survival time creates a steady production of enzymes that are required for basic cellular metabolic function at all times.

**Figure 5:**
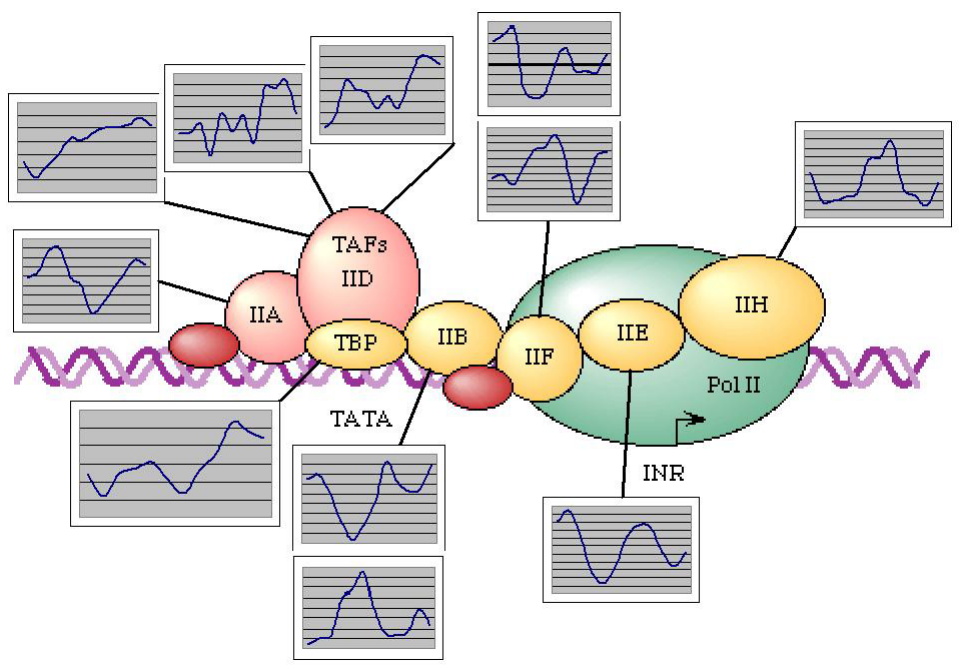
Circadian oscillation of the elements of basal transcription complex of *Arabidopsis thaliana *(Davis data set). All elements of transcription are expressed in oscillating pattern including TATA-binding protein (TBP).

While all genes oscillate, all genes are not necessarily specifically regulated to create oscillation. Also, oscillation is not likely determined by the function of each particular gene. In the dynamic cellular environment, all components experience a baseline oscillation expression rate, and the transcript abundance of each gene at any given time is relative to some other genes. These genes are also oscillating. The presence of a fraction of constantly expressed non-oscillating genes is unlikely [18]. Oscillation is simply imposed on all genes, modulating every cellular process. The illustration of this point is presented in Figure 5. The expression profiles for the major components of the basal transcription machinery (picture template taken from KEGG) are also traced to their respective probe sets in the Davis data set. Since the microarray data carries a lot of stochastic technical variation, the profiles may deviate from the ideal cosine curve. However, the circadian pattern with two peaks over two days is clearly visible in the majority of plots and is at least consistent with the others. Notably, even the TATA-binding protein expression is explicitly circadian, which is bound to affect many other transcripts.

The data obtained in two independent studies that were conducted at different times and in opposite hemispheres of the globe are very similar in the general pattern but exhibit some differences in the observed phase and the amplitude of some genes. The experimental design, though described in different words, is almost identical. Differences may be possible outside the methods described in the published papers, but the only significant differences seem to be in the time selected for the starting point (ZT, zeitgeber time) at subjective dawn, although not large, and the technique used to quantify expression values for microarrays. The Davis data set also has lower overall intensity and twice as many genes that are deemed absent as compared to the Warwick data. The latter can possibly explain the difference in signal to noise ratio between almost identically designed experiments. In both studies, the attempt to stop oscillation in the entire transcriptome by removing environmental oscillation (i.e., light) proved futile. In animal studies, changing the lighting regime from oscillating to constant darkness or dim light creates asynchrony between feeding, sleeping, and other activity patterns. As a result, a significant number of transcripts loose synchronization and identifying the baseline oscillation becomes more challenging. From a glance, the plant transcriptome data that was collected in constant light looks like the mouse transcriptome data that was collected under normal conditions with no alternation in the environment. Unfortunately, we do not have plant data that was collected under normal lighting for comparison. However, the robust oscillation under constant light in both the Davis and Warwick data leaves little space for a change. Leveling a single rhythmic environmental factor makes little impression on the pattern of gene expression in plants.

The authors of the publications that contribute to the Davis and Warwick data sets are referencing one another's works and are aware of some discrepancies, particularly in the number of rhythmic or "circadially regulated" genes. Covington and Harmer find a prevalence of higher than 10% of circadially-regulated genes intriguing and thus possible. However, neither of these research teams allowed for the possibility of all genes being expressed in circadian rhythm. This finding undermines the results of the studies that follow the separation of a small portion of rhythmic transcripts through both the analyses of over-represented pathways and the role of the molecular clock in specific pathways. Much of the results and discussion presented in these papers are based on the intuitive but unfounded assumption that all genes are expressed in a steady line pattern. Unfortunately, in the light of knowing that all genes are oscillating in a circadian pattern, these findings will have to be revised. On the other hand, the circadian timeline data that were collected for these studies are invaluable. These data could be an endless source of discovery. However, the analysis should be considered from a different angle, i.e., not whether a particular gene, co-regulated genes, or pathways are circadially-regulated but how changing experimental conditions affects oscillating properties, such as the phase and amplitude of the genes.

## Materials and methods

### Circadian expression data

#### UC Davis data set

Col-0 ecotype seeds were stratified at 4°C for 4 days before transfer to a growth chamber (22°C). Seedlings were entrained in 12-h white light (light source was cool white fluorescence tubes)/12-h dark cycles for 7 days before being released into free-running conditions of continuous white light at 22°C. Starting at subjective dawn of day 9, tissue was harvested every 4 h over the course of the next 44 h. Following standard protocols labelled cRNA targets were prepared from total RNA and hybridized to Affymetrix Arabidopsis expression GeneChips. Expression values were estimated at CSU from the original CEL files provided by the authors using dChip-derived Model-Based Expression Index.

#### University of Warwick data set

Wild-type Col-0 seedlings were used for the microarray circadian time-course experiment. Seedlings were placed immediately into LD 12:12 and grown for 7 days at 22°C. At dawn on the 8th day, they were placed into constant cool white fluorescent light. Samples were taken over two circadian cycles at 4-h intervals starting from ZT26. Samples were assayed on the Affymetrix GeneChip oligonucleotide ATH1 array (Affymetrix) according to the manufacturer's instructions. Background correction and normalization and gene expression analysis of the array data were performed using the GC-RMA routine [21] in GeneSpring version 7.2 (Silicon Genetics). The resulting table of gene expression values was downloaded from the GEO database.

### Algorithms

#### Data pre-processing

Profiles have been smoothened by a 3^rd ^degree polynomial procedure and median-subtracted. For smoothing we use seven-point Savitzky-Golay algorithm [24]. To take advantage of all points in the time series a single-pass smoothing has been applied in a circular manner, with the last points contributing to smoothing the starting points. For better compatibility, the same smoothing and median subtraction procedure has been applied to all data sets.

#### Spectral analysis

For purposes of spectral analysis, consider a series of microarray expression values for gene *x *with *N *samples of the form

*Y *= *x*_0_, *x*_1_, *x*_2_, *x*_*N*-1_

This series can be converted from time-domain, where each variable represents a measurement in time to a frequency domain using Discrete Fourier Transform (DFT) algorithm. Frequency domain representation of the series of experiments is also known as periodogram, which can be denoted by *I*(*ω*) :

$$I(\omega )=\frac{1}{N}{\left|\sum_{t=0}^{N-1}x_{t}e^{\left(-i\omega t\right)}\right|}^{2},\omega \in \left[0,\pi \right]$$

If a time series has a significant sinusoidal component with frequency *ω *∈ [0, *π*], then the periodogram exhibits a peak at that frequency with a high probability. Conversely, if the time series is a purely random process (a.k.a "white noise"), then the plot of the periodogram against the Fourier frequencies approaches a straight line [25].

#### Fisher's g-test

The significance of the observed periodicity can be estimated by Fisher *g*-statistics, as recently recommended in [14]. Fisher derived an exact test of the maximum periodogram coordinate by introducing the *g*-statistic

$$g=\frac{\max_{k}I\left(\omega_{k}\right)}{\sum_{k=1}^{N/2}I\left(\omega_{k}\right)},$$

where *I*(*ω*_*k*_) is a *k-*th peak of the periodogram. Large values of g indicate a non-random periodicity. We calculate the *p*-value of the test under the null hypothesis with the exact distribution of *g *using the following formula:

P(g>x)=∑p=11x[(−1)pn!p!(n−p)!(1−px)n−1],

where *n *= [*N/*2] and *p *is the largest integer less than 1/*x*.

This algorithm closely follows the guidelines recommended for analysis of periodicities in time-series microarray data [14] with the exception that we applied a locally developed C++ code instead of R scripts.

#### Autocorrelation

For a given a discrete time series *Y *= *x*_0_, *x*_1_, *x*_2_, *x*_*N*-1 _the autocorrelation is simply the correlation of the expression profile against itself with a frame shift of *k *data points (where 0 ≤ *k *≤ *N *- 1, often referred as the lag). For the time shift *f*, defined as *f *= *i *+ *k *if *i+k<N *and *f *= *i *+ *k *- *N *otherwise

$$R(f)=\frac{\sum_{0}^{N-1}\left(x_{\text{i}}-\bar{x}\right)\left(x_{f}-\bar{x}\right)}{\sum_{0}^{N-1}{\left(x_{i}-\bar{x}\right)}^{2}}$$

For each time series we calculate the maximum positive *R(f) *among all possible phase shifts *f *and use tabulated 0.05 significance cutoff values for correlation coefficient. Time series that shows significant autocorrelation *R(f) *with the lag *f *corresponding to one day (6 time points) are considered circadially expressed.

#### Pt-test

Consider a time series *Y *= *x*_0_, *x*_1_, *x*_2_, ... *x*_*N*-1 _in which technical variation approaches or even exceeds the amplitude of periodic expression. In a very short time series stochastic noise often obscures periodicity. However, the periodic change of the base expression level can still be identified in spite of the high noise level. If the periodogram of the original time series *IY(ω) *contains a significant peak corresponding to a particular frequency (for example, circadian) this peak results from observation is the *Y*. A random permutation would preserve the same noise level, but not the periodicity. Let *YR *be a random permutation of the time series *Y*. Its corresponding periodogram is *IR(ω)*. After DFT a periodogram *IR(ω) *would represent only the peaks occurring by chance. However it will miss the true periodic frequencies unless permutations happen to preserve the period, for example if the rank of each point *x *in permutated series *YR *is equal *x*_*Y *_± n * *p *where *n *is a natural number and *p *is a period corresponding to a significant peak in *IY(ω)*. To avoid random re-institution of periodicity we generate *YR *by multiple shuffling of randomly selected time points *x*_*n *_⇔ *x*_*m*_, where |*n *- *m*| ≠ *p*, i.e. each shuffle is swaps time points from different phase. Comparing permutations with deliberately wiped out periodicity to the original time series we can estimate whether a particular order of observations (i.e. time series) is important. For each gene expression profile we generate two series of *min(n!,100) *random permutations. Each permutated series *YR *is transformed to the frequency domain and a single peak of the periodogram *IR(ω) *is stored. The p-value for the null-hypothesis of random nature of a particular peak of periodogram can be estimated by comparing the stored *IR(ω) *values to the observed *I(ω)*:

$$p=\frac{N_{IR(\omega )\geq IY(\omega )}}{\min(n!,100)}.$$

High *p*-value exceeding the threshold, for example 0.05, means that at least 5 out of 100 random permutations of time series produce a periodogram with the same or higher peak, corresponding to a given periodicity. Low *p*-values indicate a significant difference between periodogram *IR(ω) *preserving circadian periodicity and randomly permutated periodogram *IY(ω) *with the same level of technical variation. This difference leads to rejection of the null-hypothesis of purely random nature of variation in the original time series *Y*.

#### Phase continuum

We start with phase classification, assigning each gene a phase based on maximal correlation to an ideal cosine curve. This method is superior to assigning a phase by position of peaks only because it takes into account more data. Each profile is subjected to z-score transformation equalizing the variation between time points. For each profile autocorrelation with circadian lag (*R*_*c*_) is calculated and all profiles are sorted first by phase then by descending order of *R*_*c*_. Concatenating all profiles of the same phase with equalized range of variation (amplitude) we generate a continuous stream *C*_*ph *_of measurements containing a clear signal on one end and stochastic noise on the other. This continuum is treated with low-pass frequency filter and polynomial smoothing. We analyze each phase fraction separately to detect the point at which circadian signal deteriorates beyond p = 0.05 significance cutoff. A window W moving along the stream is transformed to frequency domain using Discrete Fourier Transform (DFT). The resulting periodogram *I*_*w *_is compared a periodogram of a randomly permutated *W*_*r *_using Kolmogorov-Smirnov goodness of fit test. Once the point at which *I*_*w *_does not differ significantly from a random periodogram *I*_*wr *_is detected, we count all original gene expression profiles that have circadian signal above the established cutoff [17].

#### False Discovery Rate analysis

this methodology often applied to reduce the number of false-positive tests is based in the assumption of independent or mildly dependent [26] hypothesis testing. However, in case of testing timeline expression profiles for periodicity independence could not be assumes for a number of reasons. First, the pattern of circadian oscillation is obvious in the great majority of expression profiles as seen on heatmaps (Figure 1, for example). Second, analysis of correlation with phase shift (also used to identify phase groups) confirms high correlation of nearly all profiles to common cosine curves. Third, living cells are known to have more than one oscillator, but these oscillators are normally synchronized to the rhythm of the circadian molecular clock, active in peripheral tissues. Testing individual expression profiles for periodicity we are looking for manifestation of the same factor, hence not independent hypothesis. For these reasons FDR correction has not been applied to reduce the number of detected oscillating genes.

## Competing interests

The author declares that they have no competing interests.
